# Food allergy knowledge, attitudes and their determinants among restaurant staff: A cross-sectional study

**DOI:** 10.1371/journal.pone.0214625

**Published:** 2019-04-24

**Authors:** Adrian Loerbroks, Susanne Julia Tolksdorf, Martin Wagenmann, Helen Smith

**Affiliations:** 1 Institute of Occupational, Social and Environmental Medicine, Center for Health and Society, Faculty of Medicine, University of Düsseldorf, Düsseldorf, Germany; 2 Department of Otorhinolaryngology and Head and Neck Surgery, University of Düsseldorf, Düsseldorf, Germany; 3 Family Medicine and Primary Care, Lee Kong Chian School of Medicine, Mandalay, Nanyang Technological University Singapore, Singapore, Singapore; Ghent University, BELGIUM

## Abstract

**Background:**

There is wide international variation regarding food allergy knowledge among restaurant staff. Further, attitudes towards food allergy remain under-researched. Insights into the independent determinants of knowledge and attitudes are limited due to lacking mutual statistical adjustment for determinants/confounders in the vast majority of prior studies. In this study we aimed to contribute novel data on the food allergy knowledge and attitudes among restaurant staff in Germany whilst also examining potential determinants of both outcomes using multivariable approaches.

**Methods:**

We collected data face-to-face from 295 staff members in restaurants in Düsseldorf, Germany. Knowledge was assessed by asking participants to name three common food allergens and to answer five true/false-statements. Seven items assessed attitudes. A total of 16 potential determinants were examined using logistic regression models with backward selection.

**Results:**

Only 30% (n = 89) of the respondents correctly named three food allergens and 41% (n = 120) attained a perfect score on the true/false statements. The vast majority expressed positive attitudes toward the need for cooperation and shared responsibilities for food-allergic customers. However, the expressed attitudes towards serving customers with food allergies and validity of customer-reported food allergies were unfavorable. Determinants of food allergy knowledge (e.g. the type of restaurant, professional roles, or levels of school education) and of unfavorable attitudes (e.g. gender) were identified.

**Conclusions:**

Food allergy knowledge was suboptimal among restaurant staff and attitudes towards customers were rather poor. While we identified some determinants, additional studies are needed to systematically examine potential determinants for targetting educational interventions in the future.

## Introduction

The prevalence of food allergy is substantial [1], affecting > 10% in Europe [2]. Often symptoms (e.g. urticaria, nausea, dyspnea) may be mild, but in a few instances symptoms are severe, with rapid onset, and may result in life-threatening anaphylaxis. A limited number of foods accounts for the majority of allergic reactions, these include peanuts, milk, eggs, fish, shellfish, wheat and soy [1, 3]. Currently there is no cure for food allergies and thus successful food allergy management is dependent on the avoidance of allergen ingestion or swift treatment in case of exposure. [1]. Avoidance of allergens is highly challenging in real-life, particularly when patients’ opportunities to exert dietary control are limited, for example when eating outside one’s own home [4] or in a restaurant. In such circumstances allergen exposure is often caused by cross-contact during food preparation or the inclusion of ingredients that cannot be reasonably expected by consumers [4]. Even when consumers communicate their dietary needs to restaurant staff appropriately, the actual provision of suitable foods remains contingent upon the knowledge, attitudes and subsequent practices of the staff. Accordingly, food allergy knowledge and attitudes among restaurant staff have attracted increasing interest, particularly as prior research suggests profound knowledge gaps. An US study found that at least one quarter of staff hold important misconceptions, for instance, that it is safe for affected customers to consume small amounts of the allergen or that heating of foods destroys allergens [5]. These worrisome findings have been reproduced in various international studies [6–10]. Notably, while poor knowledge levels have consistently been confirmed there is also large international variation of knowledge; for instance in a study from Turkey as much as 88% of the participants held at least one misconception [8]. The evidence about allergy-related attitudes is markedly sparse [11–14] but suggests some unfavorable and widely held attitudes. Examples include staff not feeling responsible to inform customers about food allergens in meals or the belief that customers’ reports of food allergy are untrue [13, 14].

In addition to restaurant staffs’ level of food allergy knowledge and the nature of attitudes, it is crucial to examine possible determinants of these outcomes. Such insights enable the identification of subpopulations with particularly poor knowledge or attitudes, who can then be targeted for interventions. Prior research into determinants has been limited in terms of its i) scope and ii) methodological approach. Existing studies have generally addressed only a few determinants, such as duration of employment in the food industry, one’s professional role (e.g. waiter, chef, manager) or confidence to provide a safe meal [5–9, 12–14] and the relationships observed have been inconsistent across studies. Moreover, estimates were not mutually adjusted and it thus remains uncertain to what extent the identified correlates qualify as independent determinants. The only exception is a recent US study [11], which addressed most of the determinants mentioned above, but these did not generally emerge from multivariate regression models as significant determinants of knowledge or attitudes [11]. This highlights the importance of mutual adjustment for potential determinants (or confounders). The US study further illustrated the relevance of examining restaurant-level factors as determinants of food allergy knowledge and attitudes (e.g. the price of the most expensive food item or the number of meals typically served per day) [11]. Such determinants have been largely unaddressed in other studies.

To summarize, i) prior research has identified largely inadequate food allergy knowledge among restaurant staff with large cross-national variation, ii) restaurant staff’s attitudes towards food allergy remain under-researched, and iii) research into determinants is largely hampered by a focus on a limited number of determinants and unadjusted estimations. Based on a sample from Germany, we therefore aimed i) to contribute novel international data on restaurant staffs’ food allergy knowledge and attitudes and ii) to examine numerous determinants of both outcomes by multivariable procedures. We also aimed to further expand the current research focus to restaurant-level variables.

## Materials and methods

### Study population

Between August and October 2017, we collected data in 15 randomly selected districts from the city of Düsseldorf, Germany. The city of Düsseldorf was selected for logistic reasons as a sampling frame as the institute where this research was conducted is located in Düsseldorf. Within each district, restaurants were randomly selected and there were no exclusion criteria with regard to restaurant characteristics (e.g. no selection by type of service). We aimed to interview at least 20 adults per district and preferably one respondent per restaurant. The targeted sample size (n = 300) was based on earlier evidence indicating that such a sample size likely provides adequate statistical power for multivariable analyses [11]. Data were collected during personal visits and by self-administered questionnaires or–in case of language problems–by personal interviews. The study coordinator (ST) was present while participants provided their data and collected restaurant-level information on site (see below). When eligible individuals refused participation we gathered non-responder information. Our study was approved by the ethics committee at the University of Düsseldorf (Study ID: 5998R).

### Food allergy knowledge and attitudes

#### Instrument development

Instruments or items applied in prior studies were used to collect data on food allergy knowledge [6] and attitudes [11, 13, 14]. We refined the devised instruments based on ten cognitive interviews with restaurant staff working in varying professional roles and different types of restaurants. During those interviews, we initially explored knowledge and attitudes by open-ended questions in order to test the completeness of our instruments. Next, we explored how the items measuring knowledge and attitudes were understood and to what extent they were perceived to be relevant.

Knowledge was assessed by two previously used tests. [6] The first test assessed allergen-specific knowledge by asking participants to write down three common food allergens. Correctness of responses was evaluated based on the specification of allergens in the EU food allergen labeling regulations [15]. One point was assigned for each correctly stated allergen and thus the potential total score ranged from 0 to 3. The second knowledge test measured “general food allergy knowledge” by five true/false statements (see Table 1). The English-language instrument [6] was translated into German by the study team. Participants obtained one point for each correct answer and we calculated a total knowledge score across items.

**Table 1:** Correct responses to statements assessing general food allergy knowledge.

Item	n (%) with correct answer
Customers with food allergies can safely consume a small amount of that food (false)	243 (82.37)
Cooking, for example frying, can stop food from causing allergies (false)	247 (83.73)
A food allergy reaction can cause death (true)	266 (90.17)
If a customer is having an allergic reaction they should be served cold water to dilute the allergen (false)	193 (65.42)
Removing an allergen from a finished meal, e.g. removing the nuts, may be all that is necessary to provide a safe meal for a food allergy customer (false)	244 (82.71)

To devise a questionnaire capturing food allergy attitudes the study team first systematically searched and reviewed the instruments previously used [11, 13, 14]. We then selected items considered to measure different aspects of attitudes and translated those into German. We further developed ten additional items.

#### Cognitive interviews

The cognitive interviews suggested that the translated general knowledge test was well understood and that there were no major misbeliefs that remained unassessed. Moreover, based on the cognitive interviews the pool of attitude items developed by the study team was reduced from ten to two items. The cognitive interviews further confirmed that attitude items covered all relevant attitudinal elements, that the items were understood and that they were considered relevant by respondents. The final instrument measuring attitudes contained seven items (see Table 2). Items were presented as statements and respondents were asked to indicate whether they agreed or disagreed. This binary response option was preferred by cognitive interview participants.

**Table 2:** Responses to items measuring attitudes towards food allergy.

Item	n (%) agreeing
Service staff should be knowledgeable about food allergies	285 (96.61)
Kitchen staff should be knowledgeable about food allergies	287 (97.29)
It is my responsibility if people with food allergies have allergy reactions at my premises	197 (67.24)
I believe some food allergies indicated by the customers are not true	122 (41.78)
It is customers’ responsibility to express their food allergy needs	270 (91.53)
I would prefer not to serve customers with food allergies	54 (19.05)
The entire restaurant staff must collaborate closely to meet the needs of customers with food allergies	279 (94.58)

### Sample characteristics and potential determinants

We collected additional data on 20 characteristics and 18 of these were examined as potential determinants (see section “statistical analyses”) of food allergy knowledge and attitudes. Details on the measurement of those variables and how they were used in statistical analyses (e.g. as continuous or categorized variables) are summarized in Table 3. Our data included both individual-level data and restaurant-level data. Individual-level data comprised demographic information (age, sex, and education), occupational data (employment scheme, years of employment in the food industry, professional role, job satisfaction and two core components of burnout), and additional food allergy-related data (the potential wish for further information on food allergies, the type of preferred format to receive such information, prior participation in food allergy training, and confidence in providing an allergy-friendly meal). Restaurant level data included the number of staff members, the service type, the restaurant type, the number of tables, the most expensive main course, the price for a small glass of sparking water, and whether or not allergens were labeled in the menu. Among non-responders, we recorded their stated reason to decline participation, the observed gender, the type of service and restaurant (see above).

**Table 3:** Characteristics of study participants and potential determinants^a^ of food allergy knowledge and attitudes.

**Individual-level data**			
Age, mean, standard deviation (SD)		36.62	13.00
Sex, n, %	Men	183	62.03
	Women	112	37.97
Education^b^, n, %	None/Low/intermediate	104	39.25
	High	161	60.75
Employment scheme^c^ n, %	Full-time	196	66.44
	Part-time or marginal	99	33.56
Years in food service industry, mean, SD		12.76	11.68
Professional role, n, %	Waiter	142	48.14
	Chef	47	15.93
	Manager	86	29.15
	Multiple roles	20	6.78
Participated in food allergy training, n, %	Yes	135	45.76
	No	160	54.24
Desires further information on food allergies, n, %	Yes	145	50.17
	No	144	49.83
Format preferred for food allergy information, n, %	Brochure only	44	30.34
	Internet only	46	31.72
	Training only	27	18.62
	Multiple formats	24	16.55
Confidence in the ability to serve an allergy-safe meal	Very confident	96	32.54
	Fairly confident	102	34.58
	Confident	64	21.69
	Fairly unconfident	27	9.15
	Very unconfident	5	1.69
Overall job satisfaction^d^, n, %	Very dissatisfied	31	10.51
	Dissatisfied	15	5.05
	Satisfied	152	51.53
	Very satisfied	97	32.88
Burnout^e^, mean, SD	Emotional exhaustion^d^	15.14	5.62
	Cynicism^e^	9.25	4.16
**Restaurant-level data**^f^			
Total number of staff members, n, %	1–5	91	33.83
	6–10	95	35.32
	≥ 11	83	30.86
Type of restaurant ^g^, n, % ^a^	Full service	183	62.03
	Partial service	52	17.63
	Diner /takeaway	60	20.34
Type of food ^h^, n, %	Asian	30	10.17
	Turkish	18	6.10
	Italian	37	12.54
	International	131	44.41
	Mediterranean	13	4.41
	German	42	14.24
	Other	24	8.14
Number of tables, mean, standard deviation (SD)		13.78	10.64
Most expensive main course (€), mean, SD		15.08	8.54
Price for a small glass of sparking water (€), mean, SD		2.13	0.67
Labeling of allergens in the menu, n, %%	Yes	83	28.14
	No	212	71.86

^a^ All the listed variables were examined as determinants except for restaurant type and the preferred format for food allergy information whose cell numbers were too small. Thus, a total of 18 variables were examined as potential determinants.

^b^ Highest school degree. Low/intermediate = “Kein Abschluss/ Haupt-/Volksschule”or “Realschule“; high = “Fachhochschulreife/Abitur“.

^c^ Marginal employment (so-called “Mini Job“): salary <450€ /month

^d^ Overall job satisfaction was assessed by the question “how satisfied are you with your work?”

^e^ Burnout was measured by its two core components of burnout, i.e., “emotional exhaustion” (4 items, Cronbach’s alpha = 0.90) and “cynicism” (5 items, Cronbach’s alpha = 0.69) by the German version of the Maslach Burnout Inventory–General Survey (see: Büssing A, Glaser J. Managerial stress und burnout. A Collaborative International Study (CISMS). Die deutsche Untersuchung (Bericht Nr. 44). München: Technische Universität, Lehrstuhl für Psychologie; 1998), which we partly adapted for restaurant staff and which we pretested. Items were presented as statements and responses were provided on a 6-point Likert scale ranging from never (scored as 1) to very frequently (= 6). We averaged scores across the respective items to calculate exhaustion and cynicism subscores. The potential score ranges were 4 to 24 point s (exhaution) and 5 to 30 points (cynicism).

^f^ Restaurant-data was gathered by the study coordinator on site, except for the estimated numbers of staff members which was reported by the study participant.

^g^ Full service = orders made seated at table and food being served at table; partly service = orders are either made seated at table or food is being served at table; diner/takeaway = both order and pick-up of food at counter.

^h^ Other included, amongst others, Mexican and Indian

### Statistical analyses

We first compared characteristics of responders and non-responder using chi-squared tests. We then produced descriptive statistics for the study participants and restaurants, including the characterization of food allergy knowledge and attitudes. Next, we ran logistic regression models to examine associations between the above-mentioned potential determinants (i.e. the independent variables; except for restaurant type and the preferred format for food allergy information due to small cell numbers) and allergen-specific knowledge, general food allergy knowledge, and attitudes (i.e. the dependent variables). In doing so, we dichotomized the allergen-specific knowledge score into “adequate” (i.e. three correct allergens) versus “inadequate” (i.e. the remainder). General food allergy knowledge was dichotomized in a corresponding fashion (i.e. the perfect score of 5 points versus lower). The seven attitude items were analyzed individually. Further, as reported below in more detail, the endorsed level of agreement was very high (≥ 92%) for four out of the seven attitude items. Items with such little variation are unlikely to provide meaningful insights in association analyses and therefore we decided to examine only the three remaining attitude items as outcomes in separate logistic regression analyses. To identify potential independent determinants of knowledge or attitudes we ran logistic regression models with backward selection (using alpha = 0.05 as a threshold) to estimate odds ratios (ORs) with corresponding 95% confidence intervals (CIs). This process was repeated until no further variables could be excluded. All analyses were carried out using SAS.

## Results

### Non-responder analysis

We approached a total of 460 staff members in restaurants and 300 (65.2%) of these participated. Five participants had to be excluded as they reported in the questionnaire to be younger than 18 years. Reasons for non-participation (recorded from n = 159) related mainly to a lack of time (reported by 47.2%, n = 75), lack of interest (25.8%, n = 41) or to the fact that the manager was absent and could not be asked for permission to participate (16.4%, n = 26). Language problems were a relatively infrequent reason for non-response (10.7%, n = 17). Gender was not related to participation (p = 0.49). However, non-participants were less likely to work in restaurants with partial service and more likely to work in a diner/takeway as compared to participants (p = 0.03). Participation was also related to the restaurant type (p = 0.03). In particular, working in an Asian restaurant was related to declining to participate (17.5% among non-participants vs 10.2% among participants) while employment in an international (or mixed menu) restaurant was more frequent among participants (44.4%) than non-participants (31.9%).

### Characteristics of study participants and restaurants

Table 3 shows characteristics of the 295 study participants from 274 restaurants. Participants were in their mid-30s, but age varied considerably (standard deviation [SD] = 13.0). Roughly 38% (n = 112) were women. Educational levels were fairly high (e.g. 60.8% [n = 161] had attained the highest school degree). About two thirds (n = 196) worked full-time in the food service industry, and the mean working history in this sector equaled 12.8 years (SD = 11.7). All professional roles were represented with the most frequent being waiters (48.1%, n = 142). Approximately 46% (n = 135) of the respondents reported to have previously participated in food allergy training and 50.1% (n = 145) wished for further information on food allergies. The majority of participants (88.8%, n = 262) expressed confidence in their ability to provide allergy-friendly meals. Job satisfaction was high (84.4%, n = 249) and reflecting on the range of scores burnout levels were modest.

We included participants from restaurants of differing sizes in terms of team size and the number of tables. Most restaurants provided full-service (62.0%, n = 183) and had international menus. Prices for the most expensive main course varied widely: based on the mean value and SD, the price for the most expensive main course in about 68% of the restaurants ranged between € 6,54 and € 23,62. Food allergens were labeled in the menus of only 28.1% (n = 83) of the restaurants.

### Characterization of food allergy knowledge and attitudes

A total of 54 participants (18.3%) were unable to name any correct food allergen. One, two and three correct food allergens were reported by 14.9% (n = 44), 36.6% (n = 108), and 30.2% (n = 89) of the participants, respectively. As shown in Table 1, at least 80% of the participants provided correct answers to four of the five questions assessing general food allergy knowledge. The most frequent misbelief entailed that customers should be served water in case of an allergic reaction (correctly identified as false by 65.4% or n = 193). The total knowledge score, based on five questions,was skewed towards an elevated number of correct responses but only 40.7% (n = 120) of the participants attained the perfect score.

Attitudes (see Table 2) were favorable in terms of the norm that staff should be knowledgeable of food allergies. Also, positive attitudes were expressed towards the need for cooperation and the shared responsibility of staff and customers to enable adequate dealing with food allergies. However, negative attitudes did exist regarding customers’ reporting of food allergies, which 42% (n = 122) of the respondents believed often not to be true. As much as 19% (n = 54) of the participants specified that they would prefer not to serve customers with food allergies.

### Determinants of food allergy knowledge and attitudes

The results from the logistic regression models with backward selection are shown in Table 4. Models initially included the above-mentioned 18 potential determinants (i.e. independent variables) and were run separately for food allergen knowledge (i.e. three correct allergens versus less), general food allergy knowledge (five correct responses versus less), and the three attitude items that showed reasonable variation (i.e. not feeling responsible if people with food allergies have allergy reactions; preferring not to serve customers with food allergies; believing that some allergies indicated by customers are not true). The odds of having correctly named three allergens were lower among staff in diner/takeaways as compared to those working in restaurants with full service (OR = 0.35; 95%CI = 0.16,0.79) and higher in managers versus waiters (OR = 1.93, 95%CI = 1.08,3.46). General food allergy knowledge among staff increased with the number of tables in a restaurant (with odds increasing by 3% per additional table) and was elevated in staff with the highest school degree as compared to those with lower degrees (OR = 1.98, 95%CI = 1.16,3.37). We observed that a lack of confidence to provide a allergy-safe meal was associated with all three attitude item, that is, with the preference not to serve customers with food allergies (OR = 1.37, 95%CI = 1.01,1.84), with a sense of lacking responsibility for allergic reactions of customers at one’s restaurant (OR = 1.35, 95%CI = 1.05,1.73), and with a reduced odds of believing that customer-reported food allergies are not true (OR = 0.75, 95%CI = 0.58,0.97). Additional observations were that the wish for further information on food allergies was associated with reduced odds of the preference not to serve customers with food allergies (OR = 0.45, 95%CI = 0.23,0.87) and that women were less inclined than men to believe that customers’ report of food allergies untrue (OR = 0.57, 95%CI = 0.34–0.96).

**Table 4:** Determinants of food allergy knowledge and attitudes (logistic regression with backward selection).

Exposure	Outcomes	
	High allergen-specific knowledge	
	OR	95% CI
Diner /takeaway vs full service (reference)	0.35	0.16, 0.79
Manager vs waiter (reference)	1.93	1.08, 3.46
	High general food allergy knowledge	
	OR	95% CI
Number of tables	1.03	1.00*, 1.05
Highest school degree vs None/Low/intermediate (reference)	1.98	1.16, 3.37
	Not agreeing to be responsible when customers have allergic reaction at the premise	
	OR	95% CI
Not confident to provide a safe meal vs confident (reference)	1.35	1.05, 1.73
	Would not like to serve customers with food allergies	
	OR	95% CI
Desires further information on food allergies vs not (reference)	0.45	0.23, 0.87
Not confident to provide a safe meal vs confident (reference)	1.37	1.01, 1.84
	Belief that many of the customer-reported food allergies are not true	
	OR	95% CI
Female vs male (reference)	0.57	0.34, 0.96
Not confident to provide a safe meal vs confident (reference)	0.75	0.58, 0.97

Abbreviations: CI = Confidence interval^;^ OR = Odds ratio

* p-value < 0.05

## Discussion

This present study suggests that knowledge levels among restaurant staff are suboptimal. Attitudes were generally positive–except for attitudes towards serving customers with food allergies and the validity of customer-reports of food allergies. Given the large number of potential determinants that we examined, we found a rather limited number of significant associations, whether these are meaningful in terms of their implications for preventive action, will be discussed below.

### Knowledge levels in light of prior research

In our study, 30% of the participants were able to correctly name three food allergens. This prevalence is within in the range of estimates from prior studies that used the same measurement instrument [6–8]. In those studies, corresponding prevalences between 25% [7] and 56% [6] have been reported. We further observed that 41% of our participants attained the perfect score on the general food allergy knowledge test. Again, this observation is consistent with the previous literature. Levels of general food allergy knowledge varied widely between earlier studies; in a study from Turkey [8] and a study from the US [5] fewer respondents attained the full score (12% and 22%, respectively). The corresponding prevalences have been higher in two studies from the UK, but also varied substantially, i.e., between 33% [7] and 59% [6]. Future studies are needed to improve our understanding of the causes of these largely differing estimates across international studies. As the studies alluded to above employed the same instruments to assess knowledge levels, measurement approaches are unlikely to serve as a meaningful explanation. Differing knowledge levels may however be partially explained by sample differences; in particular when individuals with characteristics that are associated with better knowledge are making up a large proportion of the study sample (e.g. a higher proportion of managers in the sample resulting in better knowledge levels in the entire sample). However, as data on independent determinant remains sparse (see below), additional data is required to gain a better understanding of potentially relevant sample differences.

With regard to individual knowledge items, the misbelief that water ingestion may dilute food allergens was particularly common in our study (i.e. believed to be true by 35%). This particular misbelief displayed similar or higher prevalences in previous studies (between 34% [5] and 60% [7]) and was also the most common misperception in some of those studies [6, 7]. There are no established cut-offs to define (in)sufficient food allergy knowledge. However, it may reasonably be suggested that any single misconception is a cause of concern among professionals who are handling food for a food-allergic customers. This is particularly true when one assumes that (lack of) knowledge governs the displayed behavior and when the behavior has dramatic implications (e.g. exposing customers to allergens or delaying medical treatment). In light of this view, the documented knowledge deficits are worrisome.

### Attitudes in light of prior research

With regard to attitudes, the opportunities for comparisons with previous research are limited, because the evidence remains scarce and measurement instruments largely differed between studies [11, 13, 14]. Most restaurant staff seemed to hold positive norms about the level of food allergy knowledge to be expected from staff, and acknowledged their own responsibility as well as the need for cooperation among restaurant staff and with food-allergic customers to meet dietary needs. These observations from our study are in close keeping with findings from earlier studies measuring such attitudinal elements by the same items [11, 13]. The only exception relates to the respondents’ attitude to assume responsibility whenever customers have food allergic reactions on their premises, which was reported by 65% of our study participants, but only by 33% in a study from Malaysia [14]. The item measuring that particular construct may be understood differently across populations; for instance, assuming personal responsibility may be understood as taking exclusive responsibility. An alternative explanation is that the limited readiness to assume personal responsibility may be due to the fact that there is no law in Malaysia regulating food handlers’ practices [14]. In the European Union, by contrast, such regulations are in effect [15]. Although the attitude to assume personal responsibility for food allergic reactions of customers was more favorable in our study than in the study from Malaysia, it needs to be mentioned that our finding is still concerning in absolute terms: after all, as much as one third of the restaurant staff reported not to feel responsible in case of food allergic reactions. This attitude puts food allergic customers at increased risk given that attitudes may determine actual behavior [16]. Attitudes towards the validity of customer-reports of food allergy were unfavorable, as suggested by our study and an earlier US study [13]. One may speculate that the low confidence into the accuracy of customers’ reports is due to the fact that the requests of food allergic customer are wrongfully interpreted as life-style choices or personal preferences, but not as medically indicated or a potential safety issue. This notion is consistent with the stigma experienced by individuals with food allergy or their carers (e.g. being labeled as neurotic when the food allergy is disclosed) [17]. Our study is the first to suggest that a significant proportion of restaurant staff would prefer not to serve customers with food allergies. This unfavorable attitude seems to be partly due to limited self-efficacy in terms of one’s ability to accommodate the needs of food allergic customers (see below). There may be additional motives and these need to be elucidated in future research.

### Determinants

As mentioned above, prior research has examined only few determinants and yielded mixed results. Moreover, earlier work–with the exception of Radke et al. [11]–did not rely on multivariable statistical procedures and thus was unable to identify determinants independently from other determinants or confounders. In line with Radke et al. [11], we found that staff in restaurants with less comprehensive customer service (i.e., diners or takeaways) had lower knowledge levels. Radke et al. suggested that this may be explained by higher financial resources in full-service restaurants which are able to attract and retain more knowledgeable staff [11]. In our study, knowledge levels were further elevated in managers. One may speculate that managers have more detailed knowledge related to allergen labeling regulations as the implementation of such labeling would likely be their professional duty. Moreover, knowledge was higher in staff with the highest school degree. This may be explained by the observation that higher educational levels are associated with better skills related to seeking and understanding health information in the general population [18], which may also pertain to food allergy knowledge. Also, knowledge increased with the number of tables. A plausible explanation may be that more tables imply that more orders are received and that there is thus a higher likelihood of food allergy-specific requests. A sense of lacking confidence in the ability to provide a food allergy-safe meal showed a rather inconsistent pattern of associations across the three attitude items. Additional research is needed to explain theses observations and explore the underlying psychological processes. We observed that female restaurant staff were less likely than male staff to believe that customers’ reports of food allergies were untrue. This may be due to women’s higher capacity for empathy [19] and/or their greater openness towards nutritional issues, which is reflected in healthier diets and greater nutritional competence when compared to men [20].

Finally it is worth mentioning that some important independent variables were *not* associated with knowledge or attitudes. First, it is particularly striking that there was no evidence of an association of knowledge with the previous completion of food allergy training. This observation–which is consistent with most of the available evidence [5, 6, 8, 11]–may imply that the current educational opportunities are inadequate. Thus, training resources need to be improved to actually contribute to better food allergy knowledge. Second, we found that there was no evidence of a relationship of knowledge levels with the wish for information on food allergies or the individual’s confidence in the ability to provide an allergy-safe meal. Those findings imply that participation in improved food allergy training programs should be mandatory rather than contingent upon staff perception of their trainings needs, knowledge gaps and skills. In Germany, there is currently only compulsory training for restaurant staff and food workers which addresses food hygiene. That training is required before one starts to work with foods and must be repeated every two years. Regretfully, that training does not cover food allergies and training specifically related to food allergies is not compulsory at current.

### Strengths and limitations

We have relied on a previously used instrument to assess food allergy knowledge, but we devised a novel instrument to measure attitudes. Cognitive interviews ensured that our instruments were adequately understood and that they were reasonably complete. The validity of our assessment of knowledge levels is further supported by fact that the knowledge tests were completed on site in the presence of the interviewer,which rules out the possibility that respondents searched external sources, such as the internet for correct responses. A limitation of our assessment attitudes is its potential proneness to socially desirable responding. This would imply however that our observations regarding negative attitudes (e.g. preferring not to serve food-allergic customers) represent an underestimation of the true prevalence. Finally, we collected data in a single large city in Germany. Due to our multi-stage randomized recruitment procedure and the good response rate, our data are likely representative for our recruitment area, but not necessarily for other regions.

## Conclusions

Our study suggests that food allergy knowledge levels and some attitudinal elements required improvement among restaurant staff. We identified determinants of knowledge levels and attitudes and these insights can inform the identification of the target populations that may benefit most from educational interventions. As long as knowledge gaps and partially poor attitudes exist, individuals with food allergy who are eating out are advised to be aware that food allergy knowledge among staff may be defective–and this may even hold true when staff appear to be, or communicate to be, knowledgeable. It may be helpful in this respect to equip patients with strategies that increase the likelihood that their requested are adequately understood and considered. Also, food allergic customers need to be aware that common allergens are not necessarily labeled in menus (e.g. allergens were labeled in only 28.1% of the restaurants in our study) despite the fact that EU regulations require labeling of common allergens [21].

## Supporting information

S1 File Original version of questionnaire completed by respondents. (DOCX)

S2 File Translated version of questionnaire completed by respondents. (DOCX)

S3 File Form used to collect restaurant data in the original German-language. (DOCX)

S4 File Form used to collect restaurant data in the English-language. (DOCX)

S5 File Form used for collecting non-responder data in the original German-language. (DOCX)

S6 File Form used for collecting non-responder data in the English–language. (DOCX)
