# Supplementary material for: Food allergy knowledge, attitudes and their determinants among restaurant staff: A cross-sectional study
Source: PLoS One. 2019 Apr 24;14(4):e0214625. doi: 10.1371/journal.pone.0214625 (PMC6481789; doi:10.1371/journal.pone.0214625)
Supplement: S1 File — (DOCX) [file pone.0214625.s001.docx]

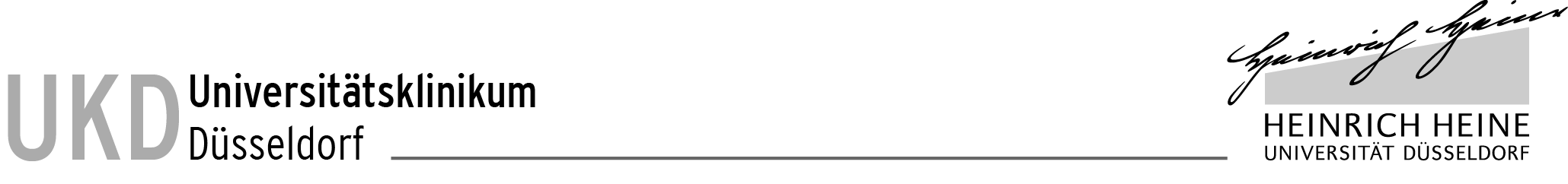


**ID:**

**Grunddaten**

| 1. Geschlecht | o Männlich o Weiblich |
| --- | --- |
| 1. Wie alt sind Sie? | ______ |
| 1. Ihr höchster Schulabschluss | o Schule beendet ohne Schulabschluss  o Haupt- oder Volksschulabschluss  o Realschulabschluss / Mittlere Reife / Fachschulreife  o Fachhochschulreife oder Abitur  o Anderen Schulabschluss (z. B. im Ausland erworben) |
| 1. Seit wie vielen Jahren arbeiten Sie in der Gastronomie? | ______ Jahre |
| 1. In welcher Position arbeiten Sie in diesem Restaurant? | o Kellner/in o Koch/Köchin o Betreiber/in |
| 1. Wie ist Ihr Beschäftigungs-umfang? | o Vollzeit o Teilzeit o Minijob |
| 1. Wie viele Mitarbeiter (inkl. Ihnen) arbeiten in diesem Restaurant? | o Vollzeit ______  o Teilzeit/Minijob ______ |
| 1. Haben Sie schon einmal an einer Schulung zu Nahrungs-mittelallergien teilgenommen? | o Ja o Nein |
| 1. Wünschen Sie sich weitere Informationen zu Nahrungs-mittelallergien? | o Ja o Nein → weiter bei Frage 11 |
| 1. Wie wünschen Sie sich solche Informationen? | o als Infobroschüre  o als Internetseite  o als Schulung  o sonstiges: ____________ |

## Berufliche Zufriedenheit

| 1. **Wie zufrieden sind Sie mit Ihrer Arbeit?** |
| --- |
| \| **Sehr unzufrieden** \| **Unzufrieden** \| **Zufrieden** \| **Sehr zufrieden** \| \| --- \| --- \| --- \| --- \| \| □ \| □ \| □ \| □ \| |

## Gesundheit

Im Folgenden finden Sie einige Aussagen zu Gefühlen, die sich auf Ihre Arbeit beziehen. Falls bei Ihnen das angesprochene Gefühl nie auftritt, kreuzen Sie bitte „Nie“ an. Falls das angesprochene Gefühl bei Ihnen auftritt bzw. Sie das Gefühl kennen, geben Sie bitte an, wie häufig das Gefühl bei Ihnen aufritt.

| Wie oft haben Sie das Gefühl: |
| --- |
| \|  \| \| **Nie** \| \| **Sehr selten** \| \| **Eher selten** \| \| **Manch-mal** \| \| **Eher oft** \| \| **Sehr oft** \| \| \| --- \| --- \| --- \| --- \| --- \| --- \| --- \| --- \| --- \| --- \| --- \| --- \| --- \| --- \| \| *Anmerkung des Studienteams:*  *In diesem Abschnitt wurden Daten zu Burnout erhoben.*  *Die verwendenden Items entstammen teilweise dem Maslach Burnout Inventar und werden daher nicht an dieser Stelle gezeigt.* \| \| □ \| \| □ \| \| □ \| \| □ \| \| □ \| \| □ \| \| |

## Nahrungsmittelallergien

Nun möchten wir Sie zu Nahrungsmittelallergien in der Gastronomie befragen.

| Bitte geben Sie an, ob Sie den folgenden Aussagen zustimmen oder nicht zustimmen: |
| --- |
| \|  \| **Stimme zu** \| \| **Stimme nicht zu** \| \| \| --- \| --- \| --- \| --- \| --- \| \| 1. Mitarbeiter im Service eines Restaurants sollten über Nahrungsmittelallergien Bescheid wissen. \| \| \| □ \| \| □ \| \| \| 1. Mitarbeiter in der Küche eines Restaurants sollten über Nahrungsmittelallergien Bescheid wissen. \| \| \| □ \| \| □ \| \| \| 1. Es liegt in meiner Verantwortung, wenn Gäste mit Nahrungsmittelallergien in meinem Lokal eine allergische Reaktion haben. \| \| \| □ \| \| □ \| \| \| 1. Am liebsten würde ich Gäste mit Nahrungsmittel-allergien gar nicht bedienen. \| \| \| □ \| \| □ \| \| \| 1. Viele, von den Gästen angegebenen Nahrungsmittelallergien sind nicht wahr. \| \| \| □ \| \| □ \| \| \| 1. Die Gäste sind dafür verantwortlich, das Servicepersonal über ihre spezielle Allergiediät zu informieren. \| \| \| □ \| \| □ \| \| \| 1. Das gesamte Restaurantpersonal muss gut zusammenarbeiten, um auf die Wünsche von Gästen mit Nahrungsmittelallergien einzugehen. \| \| \| □ \| \| □ \| \| |

| 1. Nennen Sie bitte 3 Nahrungsmittel, die Ihrer Meinung nach Allergien verursachen. |
| --- |
| \| o 1. Nahrungsmittel:  o 2. Nahrungsmittel:  o 3. Nahrungsmittel: \| \| --- \| |

## Einschätzung

| 1. **Wie sicher fühlen Sie sich, einem Gast mit Nahrungsmittelallergie ein allergie-sicheres Essen zu servieren?** |
| --- |
| \| **Sehr sicher** \| **Relativ sicher** \| **Sicher** \| **Relativ unsicher** \| **Sehr unsicher** \| \| --- \| --- \| --- \| --- \| --- \| \| □ \| □ \| □ \| □ \| □ \| |

| Bitte geben Sie an, ob Sie den folgenden Aussagen zustimmen oder nicht zustimmen: |
| --- |
| \|  \| **Wahr** \| \| **Falsch** \| \| \| --- \| --- \| --- \| --- \| --- \| \| 1. Gäste mit Nahrungsmittelallergien können ohne Probleme eine kleine Menge des betreffenden Nahrungsmittels zu sich nehmen/konsumieren. \| \| \| □ \| \| □ \| \| \| 1. Hohe Temperaturen, z.B. beim Braten, können verhindern, dass ein Nahrungsmittel eine allergische Reaktion auslösen kann. \| \| \| □ \| \| □ \| \| \| 1. Eine Nahrungsmitteallergie-Reaktion kann tödlich sein. \| \| \| □ \| \| □ \| \| \| 1. Wenn ein Gast eine allergische Reaktion hat, sollte man ihm direkt Wasser servieren, damit das Allergen im Körper verdünnt wird. \| \| \| □ \| \| □ \| \| \| 1. Um einem allergischen Gast eine sichere Mahlzeit zu servieren, reicht es aus, das Allergen von dem bereits zubereiteten Essen zu entfernen (z.B. Nüsse herunternehmen). \| \| \| □ \| \| □ \| \| |

***Vielen Dank für Ihre Teilnahme!***
