# Supplementary material for: Food allergy knowledge, attitudes and their determinants among restaurant staff: A cross-sectional study
Source: PLoS One. 2019 Apr 24;14(4):e0214625. doi: 10.1371/journal.pone.0214625 (PMC6481789; doi:10.1371/journal.pone.0214625)
Supplement: S2 File — (DOCX) [file pone.0214625.s002.docx]

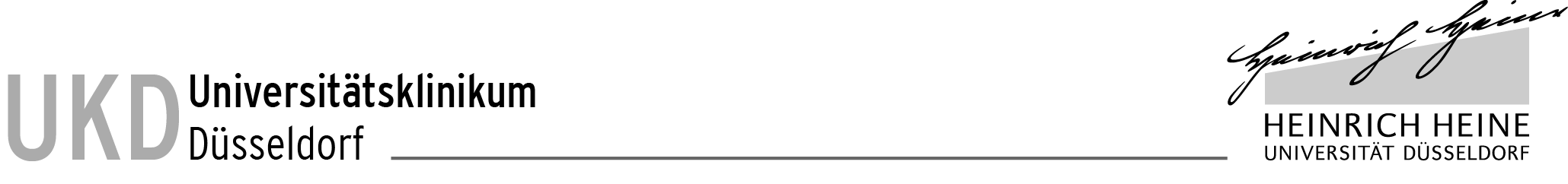


**ID:**

**Basic data**

| 1. Gender | o Male o Female |
| --- | --- |
| 1. How old are you? | ______ |
| 1. Your highest school degree | o No degree  o Low degree (i.e. Haupt- oder Volksschulabschluss)  o Intermediate degree (i.e. Realschulabschluss / Mittlere Reife / Fachschulreife)  o Highest degree (i.e. Fachhochschulreife oder Abitur)  o Other degree (e.g. not obtained in Germany) |
| 1. How many years have you worked in food service industry | ______ Years |
| 1. What is your professional role in the restaurant? | o Waiter o Chef o Manager |
| 1. What is your employment scheme? | o Full-time o Part-time o Marginal  („Mini-Job“) |
| 1. How many people (including yourself) work in this restaurant? | o Full-time ______  o Part-time/marginal ______ |
| 1. Did you ever participate in food allergy training? | o Yes o No |
| 1. Would you like further information on food allergies? | o Yes o No → Go to question 11 |
| 1. In what format would you like such information? | o Brochure  o Website  o Training  o Other: ____________ |

## Job Satisfaction

| 1. **How satisfied are you with you job?** |
| --- |
| \| **Very unsatisfied** \| **Unsatisfied** \| **Satisfied** \| **Very satisfied** \| \| --- \| --- \| --- \| --- \| \| □ \| □ \| □ \| □ \| |

## Health

| Hof often do you feel that… |
| --- |
| \|  \| \| **Never** \| \| **Very rarely** \| \| **Rather rarely** \| \| **Some-times** \| \| **Rather often** \| \| **Very often** \| \| \| --- \| --- \| --- \| --- \| --- \| --- \| --- \| --- \| --- \| --- \| --- \| --- \| --- \| --- \| \| *Comment by the authors:*  *This section collected data on burnout.*  *The items partly stemmed from the*  *German version of the Maslach Burnout Inventory and are therefore not shown* \| \| □ \| \| □ \| \| □ \| \| □ \| \| □ \| \| □ \| \| |

## Food allergies

| Please specify whether you agree or disagree with the following statements: |
| --- |
| \|  \| **Agree** \| \| **Disagree** \| \| \| --- \| --- \| --- \| --- \| --- \| \| 1. Service staff should be knowledgeable about food allergies \| \| \| □ \| \| □ \| \| \| 1. Kitchen staff should be knowledgeable about food allergies \| \| \| □ \| \| □ \| \| \| 1. It is my responsibility if people with food allergies have allergy reactions at my premises \| \| \| □ \| \| □ \| \| \| 1. I would prefer not to serve customers with food allergies \| \| \| □ \| \| □ \| \| \| 1. I believe some food allergies indicated by the customers are not true \| \| \| □ \| \| □ \| \| \| 1. It is customers’ responsibility to express their food allergy needs \| \| \| □ \| \| □ \| \| \| 1. The entire restaurant staff must collaborate closely to meet the needs of customers with food allergies \| \| \| □ \| \| □ \| \| |

| 1. Please specify three foods that cause food allergy: |
| --- |
| \| o 1. Food:  o 2. Food:  o 3. Food: \| \| --- \| |

## Self-assessment

| 1. **How confident are you in your ability to serve an allergy-safe meal to a customer with food allergies?** |
| --- |
| \| **Very confident** \| **Fairly confident** \| **Confident** \| **Fairly unconfident** \| **Very unconfident** \| \| --- \| --- \| --- \| --- \| --- \| \| □ \| □ \| □ \| □ \| □ \| |

| Please specify whether your agree or disagree with the following statements: |
| --- |
| \|  \| **True** \| \| **False** \| \| \| --- \| --- \| --- \| --- \| --- \| \| 1. Customers with food allergies can safely consume a small amount of that food. \| \| \| □ \| \| □ \| \| \| 1. Cooking, for example frying, can stop food from causing allergies. \| \| \| □ \| \| □ \| \| \| 1. A food allergy reaction can cause death. \| \| \| □ \| \| □ \| \| \| 1. If a customer is having an allergic reaction they should be served cold water to dilute the allergen. \| \| \| □ \| \| □ \| \| \| 1. Removing an allergen from a finished meal, e.g. removing the nuts, may be all that is necessary to provide a safe meal for a food allergy customer. \| \| \| □ \| \| □ \| \| |

***Thank you very much for your support!***
