# Supplementary material for: Food allergy knowledge, attitudes and their determinants among restaurant staff: A cross-sectional study
Source: PLoS One. 2019 Apr 24;14(4):e0214625. doi: 10.1371/journal.pone.0214625 (PMC6481789; doi:10.1371/journal.pone.0214625)
Supplement: S3 File — (DOCX) [file pone.0214625.s003.docx]

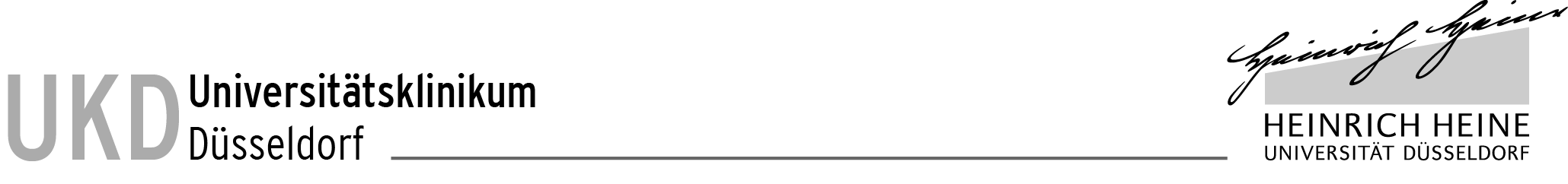


**Studie zu Nahrungsmittelallergien und Anaphylaxie: Wissen und Einstellung sowie deren Determinanten bei Gastronomie-Mitarbeitern**

**Restaurant ID:**

**Datum:**

**Dauer:**

| Restaurantküche | o Asiatisch o Mexikanisch o Deutsch  o Türkisch o International o Indisch  o Italienisch o Mediterran o sonstiges |
| --- | --- |
| Restaurant-Typ | o Vollservice o Teilservice o Imbiss |
| Anzahl der Tische im Restaurant | ______ Tische |
| Teuerstes Hauptgericht | ______ € |
| Preis 1 kleines Mineralwasser | ______ € |
| Gedrucktes Menu (mit Allergen-Kennzeichnung) | o Ja o Nein |
