# Supplementary material for: Food allergy knowledge, attitudes and their determinants among restaurant staff: A cross-sectional study
Source: PLoS One. 2019 Apr 24;14(4):e0214625. doi: 10.1371/journal.pone.0214625 (PMC6481789; doi:10.1371/journal.pone.0214625)
Supplement: S4 File — (DOCX) [file pone.0214625.s004.docx]

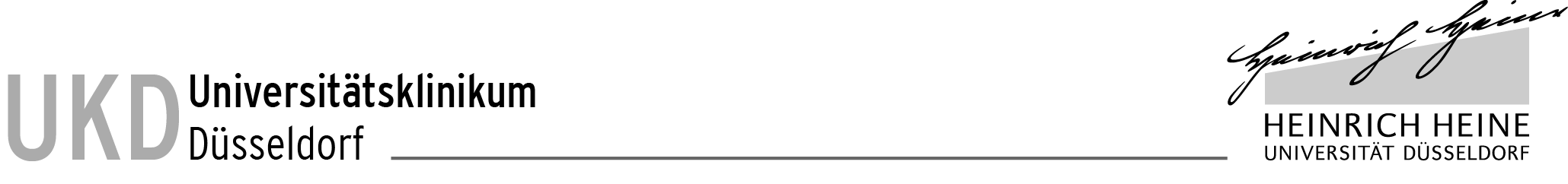


**Restaurant ID:**

**Date:**

**Duration**

| Type of food | o Asian o Mexican o German  o Turkish o International o Indian  o Italian o Mediterranean o Other |
| --- | --- |
| Type of restaurant | o Full service  o Partial service  o Dine/takeaway |
| Number of tables | ______ Tables |
| Most expensive main course | ______ € |
| Price for a small glass of sparking water | ______ € |
| Labeling of allergens in the menu | o Yes o No |
