# Supplementary material for: Food allergy knowledge, attitudes and their determinants among restaurant staff: A cross-sectional study
Source: PLoS One. 2019 Apr 24;14(4):e0214625. doi: 10.1371/journal.pone.0214625 (PMC6481789; doi:10.1371/journal.pone.0214625)
Supplement: S5 File — (DOCX) [file pone.0214625.s005.docx]

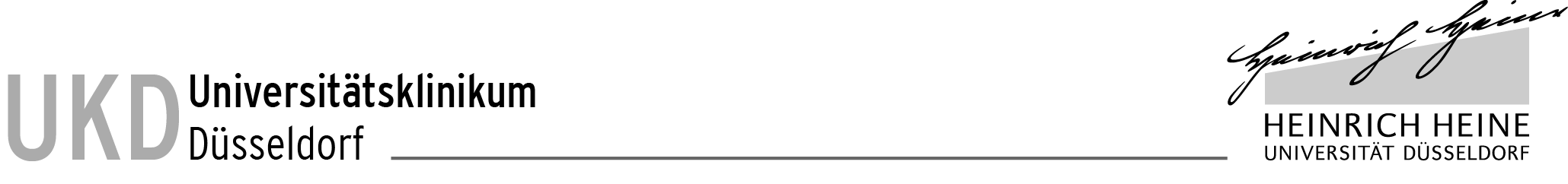


**Non-Responder Typisierung**

**Restaurant ID:**

**Datum:**

| Grund | o kein Interesse an dem Thema  o keine Zeit  o Chef/Vorgesetzter nicht da  o Sprachproblem  o sonstiges ______________ |
| --- | --- |
| Restaurantküche | o Asiatisch o Mexikanisch o Deutsch  o Türkisch o International o Indisch  o Italienisch o Mediterran o sonstiges |
| Restaurant-Typ | o Vollservice o Teilservice o Imbiss |
| Geschlecht | o Männlich o Weiblich |
